# Mechanical Behavior and Constitutive Model of Basalt-Fiber Textile-Reinforced Engineered Cementitious Composite Under Off-Axis Tension

**DOI:** 10.3390/ma19183883

**Published:** 2026-09-11

**Authors:** Zhirui An, Fahram Ayar, Shiwen Sun, Yicui Zheng, Ruihuan Wang, Yao Li, Zhu Gao, Qiangru Shen, Yanchao Wang

**Affiliations:** 1 School of Transportation and Civil Engineering, Nantong University, Nantong 226019, China; 2433320040@stmail.ntu.edu.cn (Z.A.); 2433350006@stmail.ntu.edu.cn (F.A.); 2533310009@stmail.ntu.edu.cn (S.S.); 2533320041@stmail.ntu.edu.cn (Y.Z.); whall@stmail.ntu.edu.cn (R.W.); zhugao@ntu.edu.cn (Z.G.); shenqr@ntu.edu.cn (Q.S.); 2 School of Mechanics and Civil Engineering, China University of Mining and Technology, Xuzhou 221116, China; jnliyao@163.com

**Keywords:** BTR-ECC, off-axis tension, mechanical properties, failure modes, theoretical model

## Abstract

**Highlights:**

- Strength higher at 0°/15° than 30°/45°; ultimate strain opposite.
- 0° lay-up is optimal for strength-critical and viable for energy-critical cases.
- Crack opening is 30–50 μm for all loading cases, showing excellent crack control.
- A phenomenological constitutive model well captures off-axis angle behavior.

**Abstract:**

Basalt-fiber textile-reinforced engineered cementitious composite (BTR-ECC) combines favorable durability, cost-effectiveness, and mechanics, making it a viable option for structural retrofitting. Although axial behavior is well-documented, off-axis loading zones often constitute structural weak points. To address this gap, this study experimentally and theoretically examines the mechanical responses and failure mechanisms of BTR-ECC at off-axis tensile angles of 0°, 15°, 30°, and 45°. The results demonstrate that: (1) The tensile stress–strain curves exhibit a distinct trilinear characteristic (linear elastic, strain-hardening, and fracture), featuring densely distributed microcracks confined to 30–50 μm. Dominant failure modes include textile rupture, interfacial debonding and pull-out, and PVA fiber bridging. (2) With increasing angles, tensile strength at 0°, 15°, 30°, and 45° surpasses plain ECC by 84.8%, 73.6%, 52.9%, and 54.0%, respectively, confirming effective synergistic load transfer between the basalt-fiber textile and the matrix. Despite progressive strength degradation, the strain energy density remains relatively stable across all orientations, indicating robust energy dissipation capacity. (3) A phenomenological constitutive model based on the tangent modulus approach is established to describe the off-axis tensile response. The model shows good agreement with the experimental data and may serve as a reference for the analysis of BTR-ECC structures within the calibrated range of the four tested angles.

## 1. Introduction

The cement industry is one of the major sources of global carbon emissions [1], and its energy-intensive and emission-intensive production processes pose substantial challenges to carbon reduction efforts in the construction sector [2,3]. Extending the service life of existing structures to reduce the demand for new construction has become a key strategy for lowering the life-cycle carbon emissions of the building industry [4]. In this context, textile-reinforced concrete (TRC), with its advantages of light weight, high strength, corrosion resistance, and design flexibility, has shown considerable potential in structural strengthening and life extension [5,6]. TRC is composed of high-performance textile embedded in a fine-grained mortar matrix. Its mechanical properties, particularly tensile strength and crack control capability, are highly dependent on the interfacial behavior between the yarns and the matrix, which governs the efficiency of stress transfer and the overall load-bearing capacity [7]. However, the brittleness of the mortar matrix in conventional TRC tends to cause interfacial debonding and slip of the yarns, which restricts the ductility and strength utilization of the material [5].

To overcome the above limitations, researchers have proposed using engineered cementitious composite (ECC), which exhibits strain-hardening and multiple-cracking characteristics, as a substitute for ordinary mortar, thereby establishing a textile-reinforced ECC (TR-ECC) system. This system realizes a synergistic effect combining the continuous reinforcement of the textile and the fiber-bridging toughening of the ECC matrix. The ECC matrix provides a tensile strain capacity exceeding 4% and multiple-cracking behavior, which establish a favorable basis for deformation and energy dissipation [8]. Meanwhile, the textile effectively restrains cracks, enhancing post-cracking stiffness and load-bearing capacity [9]. This enables TR-ECC to exhibit dense distributed cracking under tension, maintaining good crack control capability and sustained strain-hardening response, thus improving the ductility and damage tolerance of the structure [10,11]. However, current studies on TR-ECC have mostly focused on the mechanical properties and strengthening mechanisms under idealized axial loading, where the loading direction is parallel to the yarn orientation in the textile. For example, Zhang et al. [12] explored the uniaxial tensile behavior of basalt–glass fiber textile-reinforced ECC, along with its effectiveness in strengthening masonry columns under axial compression. Similarly, Chen et al. [13] examined the tensile properties of carbon-fiber textile-reinforced high-early-strength ECC and its confinement effect on pre-damaged concrete columns. These findings have primarily established the baseline behavior for this specific loading scenario.

However, in reality, structural members such as beam-column joints are frequently subjected to multiaxial stress states, causing the principal stress direction to misalign with the material’s principal axis and inducing off-axis loading. Due to the marked orthotropy of TR-ECC, such loading complicates the stress state within the textile reinforcement and along the yarn/ECC interface. Consequently, the mechanical response under off-axis tension can differ substantially from that under axial loading. Nevertheless, this off-axis behavior remains poorly understood. Systematic investigation into the underlying failure mechanisms is therefore required to establish a robust theoretical foundation for the engineering application of TR-ECC in complex stress environments.

Currently, some studies have been conducted on the off-axis behavior of TRC. For example, Hegger and his co-workers [14,15,16] conducted systematic tensile tests to map the stress–strain response across various angles, proposing empirical reduction factors to account for fiber misalignment. Building on this, El Kadi et al. [17] performed quasi-static and cyclic (N = 25) four-point bending tests on TRC beams oriented at 0°, 22.5°, and 45°. Their work quantified the influence of orientation on strength, post-cracking stiffness, and crack evolution, while also providing residual performance metrics post-cycling. Complementing these efforts, Ozdemir et al. [18,19] investigated the flexural properties of TRC reinforced with carbon and basalt textiles laid at 45°. However, the inherent brittleness of the mortar matrix limits the material’s performance. Once initiated, cracks propagate rapidly, preventing the full exploitation of the textile’s tensile capacity.

TR-ECC mitigates the inherent brittleness of conventional TRC mortars. Although research on its off-axis behavior remains nascent, scholarly attention is growing. Our group has contributed preliminary work in this area. Xu et al. [20] tested glass-fiber textile-reinforced ECC (GTR-ECC) under off-axis tension, characterizing how angle variations affect strength, cracking, and failure. An anomalous result showed lower tensile strength at 0° than at 15°. We attributed this to competing strengthening and weakening effects within the textile reinforcement and proposed a corresponding model. Subsequent work on carbon-fiber textile-reinforced ECC (CTR-ECC) [21] revealed a transition from brittle textile rupture under axial loading to ductile interlaminar delamination under off-axis loadings. Together, these studies confirm that off-axis loading fundamentally alters the mechanical response and failure modes of TR-ECC, limiting the applicability of data derived from standard axial tests.

However, neither study incorporated basalt-fiber textiles, which offer distinct advantages for structural strengthening. Basalt-fiber textiles not only retain the cost advantage but also exhibit good alkali resistance and durability in the alkaline cementitious environment during long-term service; moreover, they outperform glass-fiber textiles in terms of high-temperature resistance and flame retardancy [22]. Compared to carbon textiles, basalt is considerably more economical. Furthermore, as a volcanic rock derivative composed primarily of silicates, basalt textile exhibits inherent physicochemical compatibility with cementitious matrices, promoting interfacial bond stability. Consequently, the mechanical behavior of BTR-ECC or BTRM has attracted considerable research attention. Existing studies have examined the uniaxial mechanical behavior of BTR-ECC/BTRM under various loading conditions [23,24] and temperature environments [25,26], as well as their performance in structural strengthening applications [27,28,29]. For further details on basalt textiles and basalt textile-reinforced concrete, readers are referred to the review studies by Al-Kharabsheh et al. [30] and Immanuel et al. [6].

Nevertheless, existing research on BTR-ECC has predominantly focused on uniaxial mechanical performance, with few studies addressing its off-axis behavior. Although our group has investigated the off-axis mechanical behavior of GTR-ECC [20] and CTR-ECC [21], these findings cannot be directly generalized to BTR-ECC. The mechanical response of TR-ECC is highly dependent on the reinforcing textile: glass and carbon textiles yield markedly different mechanical properties and failure modes, while basalt-fiber possesses intermediate mechanical properties between the two. Consequently, the off-axis behavior and failure mechanisms of BTR-ECC remain largely unknown, warranting a systematic investigation.

In this study, the off-axis mechanical behavior and underlying failure mechanisms of BTR-ECC are investigated. A phenomenological constitutive model is also proposed to facilitate engineering applications.

## 2. Materials and Methods

### 2.1. ECC

**Figure 1:**
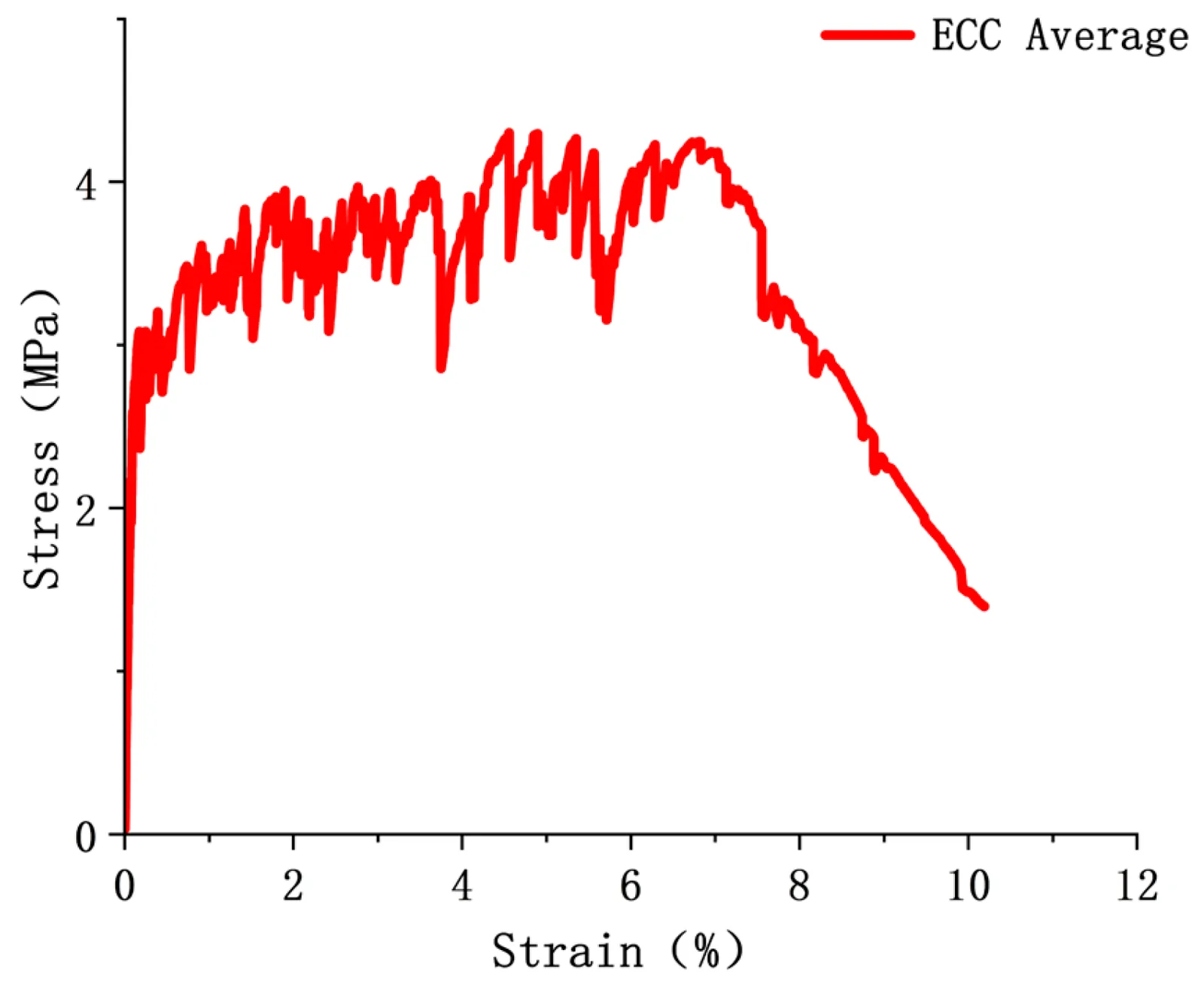
Tensile stress–strain curve of ECC (redrawn from data in [20]).

The ECC mix design, PVA fiber parameters, and curing regime are identical to those used in our prior work [20]. Both studies utilized the same batch of premixed ECC mortar and the same type and content of PVA fibers. Both the premixed mortar and the PVA fiber were purchased from Sino-Sina Building Materials Co., Ltd., Zhengzhou, China. The specimen fabrication was conducted by the same personnel to minimize human-induced variability. Although the ECC specimens were not cast concurrently with the BTR-ECC specimens in the present study, it should be noted that this constitutes a limitation, as the ECC data serve as an external reference rather than a fully equivalent contemporaneous control. Nevertheless, the high consistency in raw materials, manufacturing procedures, and testing standards ensures that the ECC data serve as a reliable benchmark. Therefore, the stress–strain curve of the ECC obtained in our previous work [20] (as shown in Figure 1) is appropriately adopted for analysis in this study. For completeness, Table 1, Table 2 and Table 3 present the chemical composition of the premixed mortar (purchased from Sino-Sina Building Materials Co., Ltd.), the physical parameters of the PVA fibers, and the mix proportions of the ECC, respectively.

As Figure 1 illustrates, the material exhibits distinct strain-hardening and high-ductility behavior. The curve begins with a brief linear-elastic rise, followed by a strain-hardening stage characterized by pronounced serrated fluctuations. Test data indicate an average first-cracking stress of 3.07 MPa and a peak strength of 4.18 MPa. Most importantly, the ultimate tensile strain reached 7.56%.

### 2.2. Epoxy-Impregnated Basalt-Fiber Textile

The basalt-fiber textile (BS-120) was purchased from Yixing Ruibang High-Performance Fiber Products Co., Ltd., Yixing, China. It featured a nominal mesh opening of 5 mm × 5 mm, an area density of approximately 120 g/m^2^, and an equivalent yarn cross-sectional area of about 0.26 mm^2^. To enhance stress transfer within the yarns and improve interfacial bonding with the ECC matrix, all textiles were impregnated with a low-viscosity epoxy resin (Product: YT-CC302S, resin-to-hardener ratio = 3:1 by weight, final resin content ≈ 55%). The procedure involved cutting the basalt fiber grid to the required dimensions, clamping one edge between two slender wooden plates, and laying it flat on a plastic sheet. The resin mixture was then applied evenly onto the fabric surface using a brush-coating method. Then, the impregnated textile was hung for curing at room temperature for 24 h.

**Figure 2:**
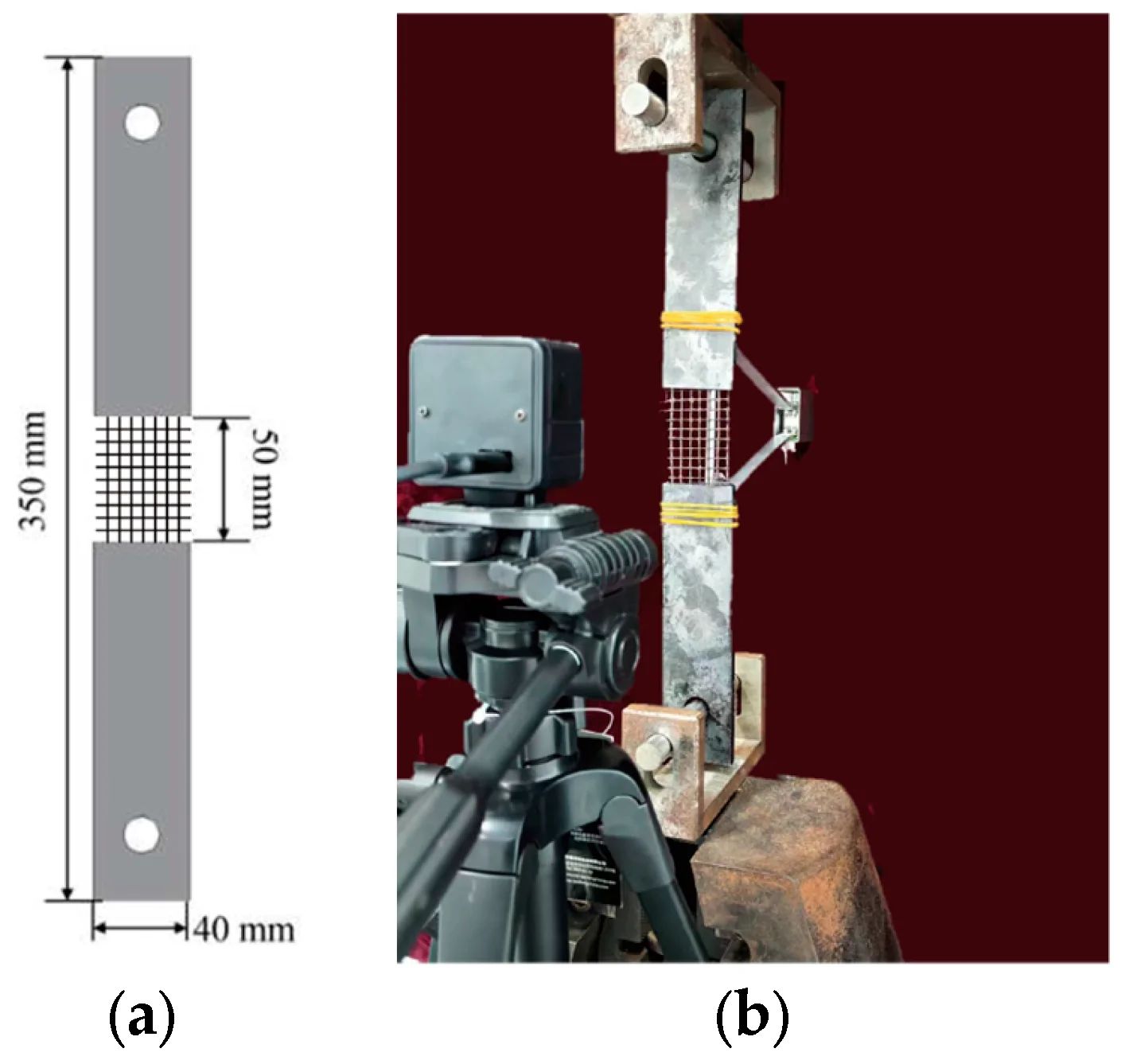
Tensile specimen of textile and test setup: (**a**) specimen dimensions; (**b**) test setup.

Once fully cured, the textiles were cut to the final specimen dimensions for tensile testing. The tensile properties were evaluated following the procedure shown in Figure 2, with specimens clamped to ensure initial straightness and loaded at a constant rate of 0.5 mm/min. An extensometer was used to measure the displacement of the textile, as shown in Figure 2b. A slight pre-tensioning operation was applied to the specimen. Owing to the high rigidity of the steel tabs and their firm epoxy bond to the textile, the effects of grip slip and machine compliance were negligible.

**Figure 3:**
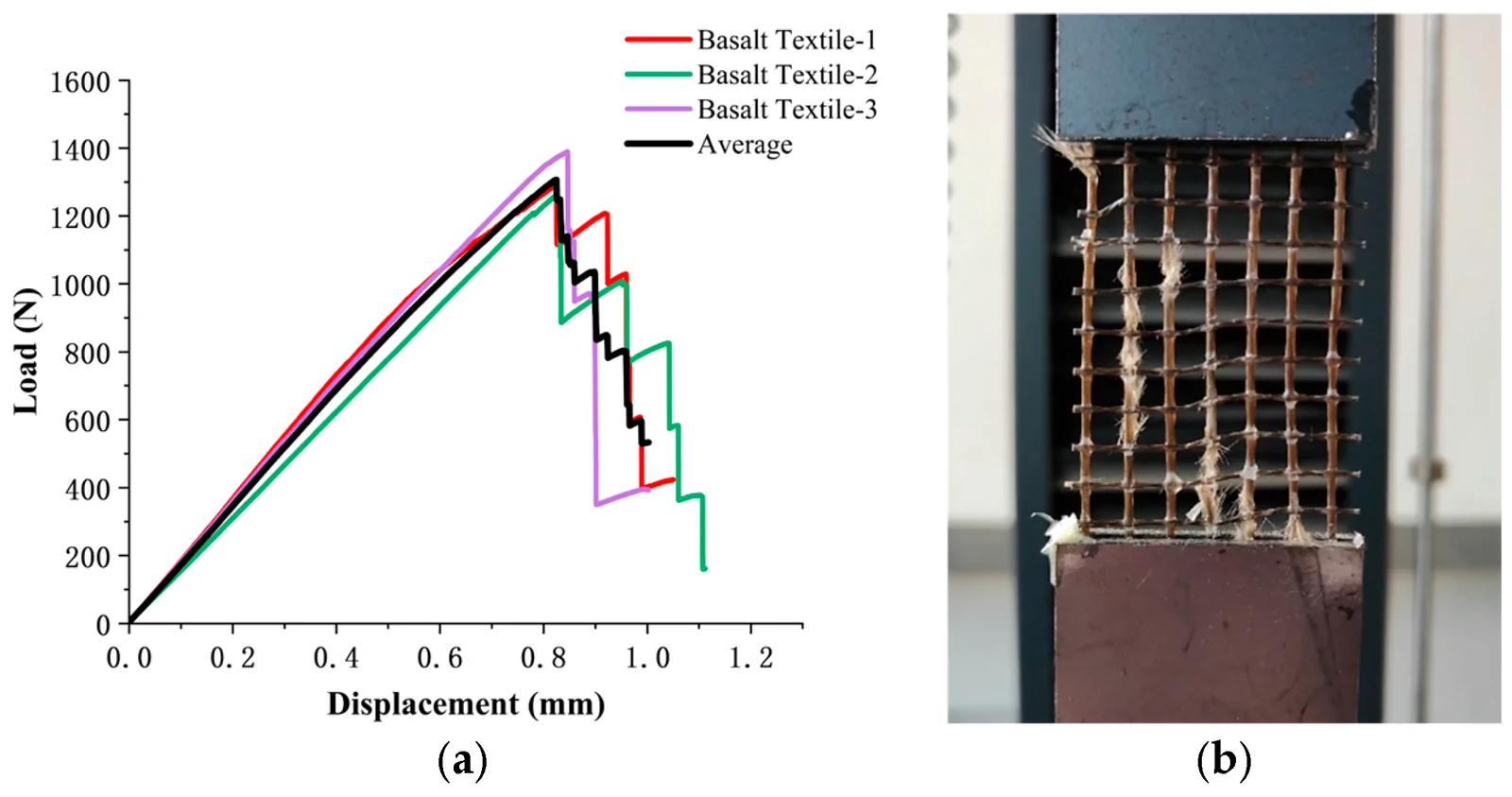
Tensile test results of basalt-fiber textile: (**a**) Tensile load–displacement curve; (**b**) Failure mode.

Uniaxial tensile test results for the basalt-fiber textile are given in Figure 3. Average mechanical properties were calculated from three valid load–displacement curves. All curves featured a linear pre-peak segment, reflecting purely elastic deformation where both warp and weft yarns shared the applied load. Beyond the peak, the curves did not follow a smooth descending path but dropped in discrete steps, as shown in Figure 3a. This stepwise response reflects progressive fracture of individual yarns. When a subset of yarns reached their ultimate strength, brittle rupture triggered a sharp load drop. Load was then redistributed to intact yarns, which continued carrying load until the next yarn failed. Measured ultimate load averaged ~1400 N at a failure displacement of ~0.8 mm, corresponding to an ultimate strain of ~1.6% (0.8 mm/50 mm = 1.6%). This aligns with reported ultimate strain values for basalt textiles (~1.82%) [31]. The resulting tensile strength and elastic modulus were approximately 769 MPa and 48.1 GPa, respectively. Due to uneven tension among individual yarns, fabric-level measurements are generally lower than single-yarn tests; however, since fabric-level results better reflect the actual stress state within the BTR-ECC composite, this testing method was adopted.

### 2.3. BTR-ECC

BTR-ECC specimens were fabricated using a layered casting technique in wooden molds. Each tensile specimen measured 300 mm long (400 mm overall including steel end-tabs), 40 mm wide, and 20 mm thick, comprising four 5 mm thick ECC layers alternating with three basalt-fiber textile layers. The textile layers were sandwiched between adjacent ECC layers, giving a spacing of approximately 5 mm between adjacent textile layers. The off-axis angle α denoted the angle between the textile warp direction and the specimen longitudinal axis, which corresponded to the loading direction. Tests were conducted at four angles: 0°, 15°, 30°, and 45°. The selected angles cover the primary off-axis loading scenarios of BTR-ECC. Limited by experimental workload, these four angles are sufficient to reveal the variation trend of mechanical response. This selection also aligns with practice widely adopted in the literature for textile-reinforced polymer or ceramic composites [32,33].

Following casting, specimens remained in their molds under standard curing conditions for 24 h prior to demolding, then continued curing until the age of 28 days. The numbers of weft yarns in the cross-section for the 0°, 15°, 30°, and 45° off-axis specimens are 8, 7.7, 6.9, and 5.7, respectively, while the corresponding warp yarn numbers are 0 (since the warp direction is perpendicular to the loading direction in the 0° specimen), 2.1, 4.0, and 5.7. The yarn numbers are averaged values based on the specimen width (40 mm). The reinforcement ratio is approximately 1.56%. The stress calculation area is defined as the full cross-sectional area of the specimen (40 mm × 20 mm = 800 mm^2^).

**Figure 4:**
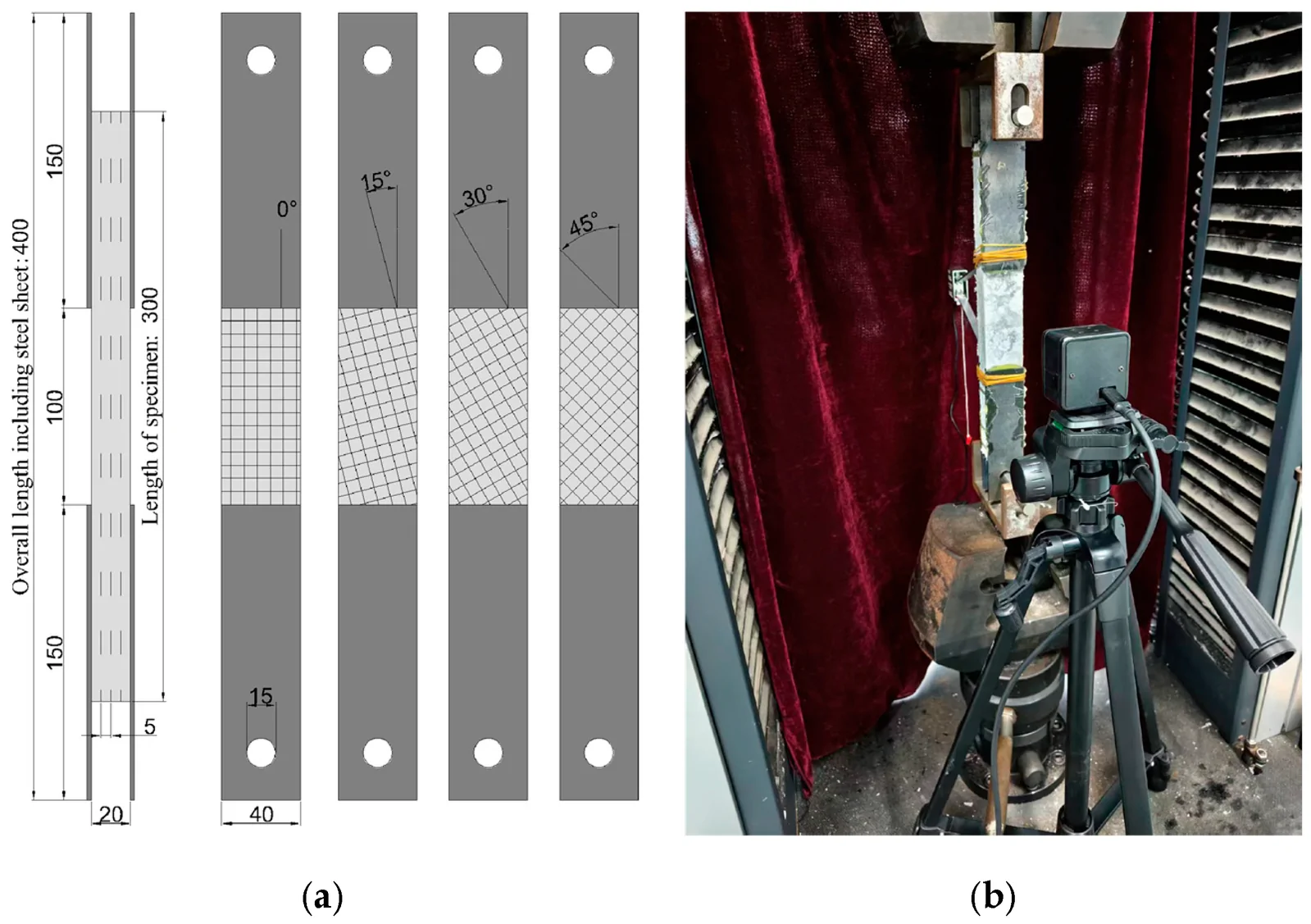
Test setup for BTR-ECC tensile tests: (**a**) BTR-ECC tensile specimens; (**b**) Installation method.

To ensure efficient load transfer and mitigate end effects from stress concentration, steel end tabs were bonded to both specimen ends using epoxy resin and left to cure at room temperature for three days (Figure 4a). The gauge length was set at 100 mm. Tensile tests were performed on a universal testing machine (MTS/SANS CMT5205, maximum capacity: 200 kN, manufactured by MTS Systems (China) Co., Ltd., Shanghai, China) under displacement control at a rate of 0.5 mm/min. Alignment was controlled using a dedicated fixture. The specimen was lightly secured in the upper grip, aligned vertically, and firmly clamped in the lower grip. The validated force measurement range provided by the manufacture is 0.4–100% of full scale, with an indication error within ±0.5%. An extensometer (Model: YYU-25/100), manufactured by Jinan Lidong Experimental Equipment Co., Ltd., Jinan, China, with a gauge length of 100 mm and a measuring range of 25 mm was used to measure the strain, offering a measurement precision of up to 10^−9^. A high-resolution digital camera (Model: KS50M-778), manufactured by Shenzhen Jinqianxiang Technology Co., Ltd., Shenzhen, China, was used to continuously capture images of the specimen surface at 5 fps, with a resolution of 8160 × 6120 pixels (Figure 4b). For each case, seven specimens were prepared and tested. Curves exhibiting fracture outside the gauge length, slippage or debonding of the steel tabs, or significant deviations from the main dataset were excluded. According to Tran et al. [34], three reproducible stress–strain curves are sufficient to effectively characterize the material’s mechanical response. Therefore, for each case, three consistent curves were selected to compute the average curve, which was used to represent the mechanical response of BTR-ECC.

Compared to our previous studies on GTR-ECC [20] and CTR-ECC [21], the basalt textile used in this study shares the same weaving pattern, nominal mesh opening size (5 mm), and resin impregnation treatment as the AR-glass and carbon textiles employed in those earlier works. Specimen dimensions, fabrication methods, and testing protocols for BTR-ECC were kept consistent with those in the prior studies. The reinforcement ratios of GTR-ECC, BTR-ECC, and CTR-ECC are approximately 0.94%, 1.56%, and 4.1%, respectively.

It is worth noting that different specimen geometries were adopted for plain ECC and BTR-ECC in accordance with relevant standards. The tensile test for plain ECC followed the work by Yokota et al. [35], which specifies dog-bone specimens to ensure uniform stress distribution. For BTR-ECC, rectangular specimens were employed according to RILEM TC232-TDT [36] and Kim et al. [37], primarily to avoid stress concentration at the grips and facilitate specimen alignment. Despite the difference in geometry, the collected data for both materials were extracted from the effective gauge length, and three reproducible stress–strain curves were obtained for each case. Furthermore, given that the loading rates and tensile testing machine were identical, the influence of specimen geometry on the measured mechanical properties is minimized, and the results remain well comparable.

## 3. Results and Discussion

### 3.1. Stress–Strain Behavior of BTR-ECC

#### 3.1.1. Overview of Experimental Data

**Figure 5:**
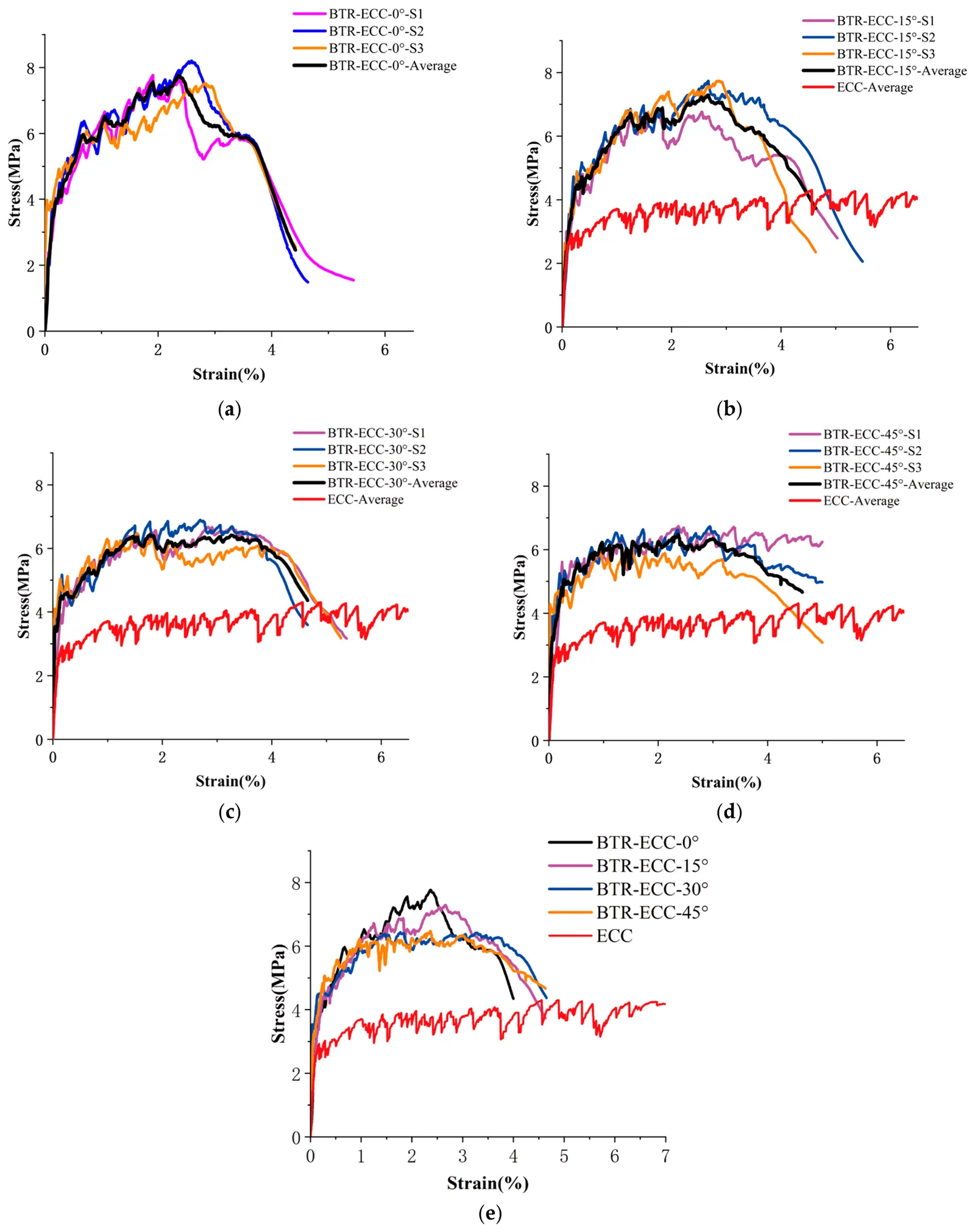
Off-axis tensile stress–strain curves of BTR-ECC: (**a**) 0°; (**b**) 15°; (**c**) 30°; (**d**) 45°; (**e**) comparison at different off-axis angles.

Figure 5a–e present the tensile stress–strain curves of BTR-ECC at various off-axis angles alongside those of plain ECC. The average curves were obtained by resampling individual stress–strain curves to a common strain domain (from 0 to the minimum ultimate strain among all valid specimens) via linear interpolation, ensuring that the averaged response remains within the experimentally captured range for all specimens. Near-zero stresses in the initial stage were retained without special exclusion, as their effect on the average curve is negligible.

Table 4 lists the mechanical property parameters of BTR-ECC obtained at each off-axis angle, including ultimate strength, ultimate strain, and strain energy density (the energy absorbed per unit volume prior to failure). Since BTR-ECC exhibits quasi-ductile rather than brittle failure, identifying an exact failure point is difficult. Therefore, the ultimate strain is determined as the strain at which the stress–strain curve starts to drop markedly and beyond which no plateau is observed. The strain energy density u was calculated according to Equations (1) and (2) with respect to the ultimate strain, where Equation (1) applies to the stress–strain curve expressed as a continuous function, and Equation (2) applies to the experimental curve represented by discrete data points.

(1) $$u^{u}=\int_{0}^{\varepsilon^{u}}\sigma \;d\varepsilon$$

(2) $$u^{u}\approx \sum_{i=1}^{k}\left[\left(1/2\right)\cdot \left(\sigma_{i}+\sigma_{i-1}\right)\cdot (\varepsilon_{i}-\varepsilon_{i-1})\right]$$

In Equation (1), $u^{u}$ is the strain energy density of the specimen, and $\varepsilon^{u}$ is the ultimate strain prior to failure. In Equation (2), i is the index of the data point, k is the index of the data point at specimen failure, and $\sigma_{i}$ and $\varepsilon_{i}$ are the stress and strain at the i-th data point, respectively.

#### 3.1.2. Data Consistency Evaluation

To assess the consistency and repeatability of the obtained experimental data, the corrected mean absolute deviation (CMAD) was adopted. For ECC-based composites exhibiting multiple cracking and strain-hardening behavior, stress–strain curves inherently fluctuate in the hardening plateau due to steady-state crack development. Traditional pointwise statistical methods (e.g., standard deviation) are highly sensitive to these intrinsic fluctuations and may amplify the apparent scatter. In contrast, CMAD evaluates curve consistency as a “shape ensemble,” offering better robustness against such inherent variations. Accordingly, CMAD was used to quantify the scatter of key mechanical parameters, while the global CMAD was employed to evaluate the consistency of the entire stress–strain curve. Their definitions are given in Equations (3) and (4).

(3) $$CMAD=\frac{1}{n}\sum_{i=1}^{n}\left|\frac{\sigma_{i}-\bar{\sigma }}{\bar{\sigma }}\right|\times 100\%$$

(4) $$Global\;CMAD=\frac{1}{mn}\sum_{i=1}^{n}\sum_{j=1}^{m}\left|\frac{\sigma_{ij}-{\bar{\sigma }}_{j}}{{\bar{\sigma }}_{j}}\right|\times 100\%$$

where n is the number of specimens, m is the number of data points per specimen, $\sigma_{i}$ is the tensile strength of the i-th specimen at a given strain, $\overset{ˉ}{\sigma }$ is the average tensile strength of the group, $\sigma_{ij}$ is the stress of the i-th specimen at strain $\varepsilon_{j}$, and $\bar{\sigma_{j}}$ is the average stress of all specimens at the same strain $\varepsilon_{j}$.

Based on Equations (3) and (4), the CMAD values of tensile strength at off-axis angles of 0°, 15°, 30°, and 45° are 3.1%, 6.0%, 3.5%, and 5.9%, respectively; the corresponding global CMAD values for the stress–strain curves are 6.0%, 8.3%, 5.3%, and 8.3%, respectively. These CMAD and global CMAD values are all at relatively low levels, indicating that the experimental data under each condition have good repeatability and consistency, and can reliably reflect the mechanical behavior of the material under these loading states.

#### 3.1.3. Analysis of Key Mechanical Properties

First, regarding the first-cracking stress, no clear trend with respect to the off-axis angle is observed, given the relatively high CMAD values across all loading cases. This considerable scatter is primarily attributed to the random distribution of constituent materials, including sand and PVA fibers. Furthermore, the mechanical advantage of the textile at 0° is offset by a more pronounced weakening effect. Specifically, the weft yarns form through-width pores in the cross-section of the ECC matrix at 0°, whereas the yarn-induced pores become elliptical under other off-axis angles. This difference in pore morphology contributes to the offset of the textile’s mechanical advantage, resulting in no distinct difference in the first-cracking stress across the off-axis angles.

The introduction of the basalt-fiber textile substantially enhances the tensile strength of the composite. Compared to plain ECC, the strength enhancement ratios of BTR-ECC at off-axis angles of 0°, 15°, 30°, and 45° are 84.8%, 73.6%, 52.9%, and 54.0%, respectively. As the off-axis angle increases, the enhancement effect exhibits a nonlinear decline: from 0° to 15°, strength decreases by only 6.1%, while the respective losses from 0° to 30° and from 0° to 45° reach 17.3% and 16.6%. This trend is directly related to the degraded off-axis tensile performance of the basalt-fiber textile itself, and also indicates the meso-mechanical mechanism by which stress transfer efficiency drops as the yarn orientation deviates from the loading direction. The results show that at small angular deviations (≤15°), the material can still retain most of its axial strength, while at large angular deviations (≥30°), more substantial but relatively stable strength losses occur. Unlike GTR-ECC, which exhibits the anomalous behavior of higher off-axis strength than that at 0°, BTR-ECC shows a normal overall decreasing trend with increasing off-axis angle. While BTR-ECC shares a similar decreasing trend with CTR-ECC, its strength reduction is far less drastic than that of CTR-ECC.

In terms of ultimate strain, BTR-ECC under off-axis loading exhibits lower values than that of the plain ECC. Moreover, small off-axis angles (0° and 15°) yield lower ultimate strains than large off-axis angles (30° and 45°). This trend may be attributed to a transition in the textile deformation mechanism: as the off-axis angle increases, the dominant loading mode of the textile shifts from axial tension to shear deformation. Since the textile can accommodate substantially greater strains under large off-axis angles than under small ones, the composite exhibits improved overall deformability at larger angles. In addition, the in-plane geometric deformation of the textile at crack locations is more pronounced for large off-axis angles than for small ones, which further contributes to the overall strain. In contrast to GTR-ECC, whose ultimate strain shows no clear trend with off-axis angle and remains within a range of 4.5–5.5%, BTR-ECC exhibits a distinct trend with off-axis angle. This difference suggests that the deformability of GTR-ECC is predominantly governed by the ECC matrix, whereas that of BTR-ECC depends on the synergistic effect between the textile and the matrix. Compared to CTR-ECC, whose ultimate strain is very low under axial loading (0°) but increases substantially at other off-axis angles (exceeding 5%), BTR-ECC shows relatively stable ultimate strains across all off-axis angles. This is because CTR-ECC undergoes brittle failure at 0° but delamination at other angles, whereas BTR-ECC maintains a good synergistic effect between the textile and the matrix across all cases.

Finally, regarding strain energy density, which serves as a comprehensive indicator of material toughness, the values for BTR-ECC at each off-axis angle range from 217 to 223 kJ/m^3^, all lower than that of the plain ECC (278 kJ/m^3^), with an average reduction of approximately 21.1%. This result can be understood from the trade-off between ultimate strength and ultimate strain. At 0°, the strength is higher but the strain is relatively low. Under off-axis loading, although the strength decreases, the ultimate strain increases substantially. These two factors counteract each other, resulting in fairly close energy absorption capacities of BTR-ECC across different off-axis angles—a trend also observed in GTR-ECC, as both materials maintain consistent failure modes for every case. CTR-ECC, however, shows a markedly different pattern. At 0°, its strain energy is much lower than that of BTR-ECC and BTR-ECC, where brittle failure limits deformability. At other off-axis angles, delamination occurs. This substantially enhances deformation capacity. Although the strength of CTR-ECC decreases from its 0° value, it still exceeds that of BTR-ECC and GTR-ECC. As a result, its strain energy far exceeds that of both.

Overall, the experimental results demonstrate that the tensile strength of BTR-ECC generally decreases with increasing off-axis angle (with the values at 30° and 45° being very close), while the strain energy density remains relatively stable at approximately 220 kJ/m^3^ across all tested angles. These findings offer clear guidance for practical engineering applications. For strength-critical scenarios, a 0° lay-up should be prioritized to achieve optimal strengthening performance. Meanwhile, for energy-absorption-critical scenarios, the 0° lay-up also delivers good performance. Furthermore, from a construction tolerance perspective, since the tensile strength decreases by only about 6.1% from 0° to 15°, it is recommended that the loading direction be kept as parallel to the warp direction as possible in BTR-ECC-strengthened structures, but minor off-axis angles (<15°) are considered acceptable in practice, as their effect on structural performance is within an acceptable range.

### 3.2. Failure Modes and Mechanisms

#### 3.2.1. Overview of Failure Morphology

**Figure 6:**
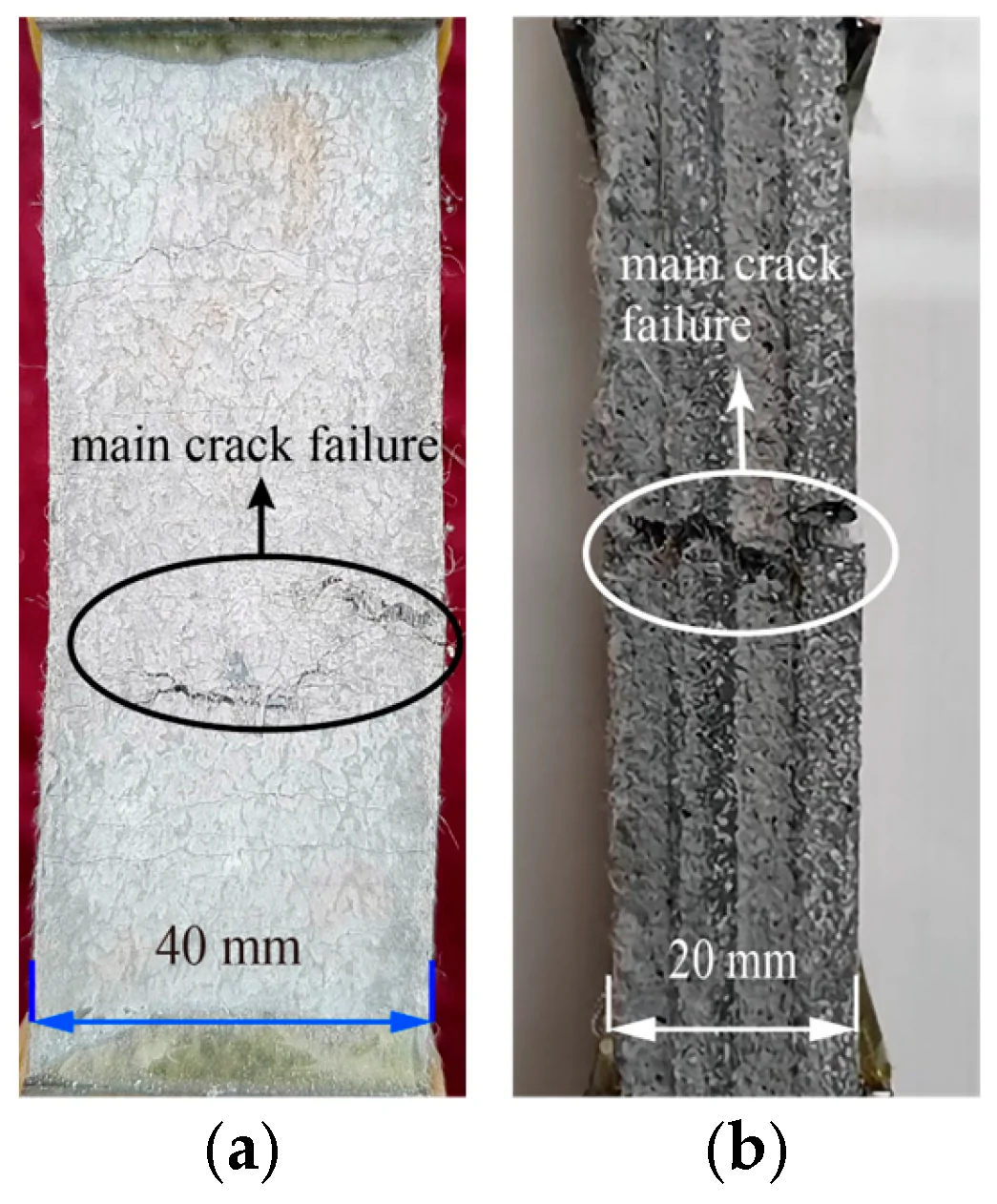
Failure mode of BTR-ECC at 0° off-axis angle: (**a**) front view; (**b**) side view.

**Figure 7:**
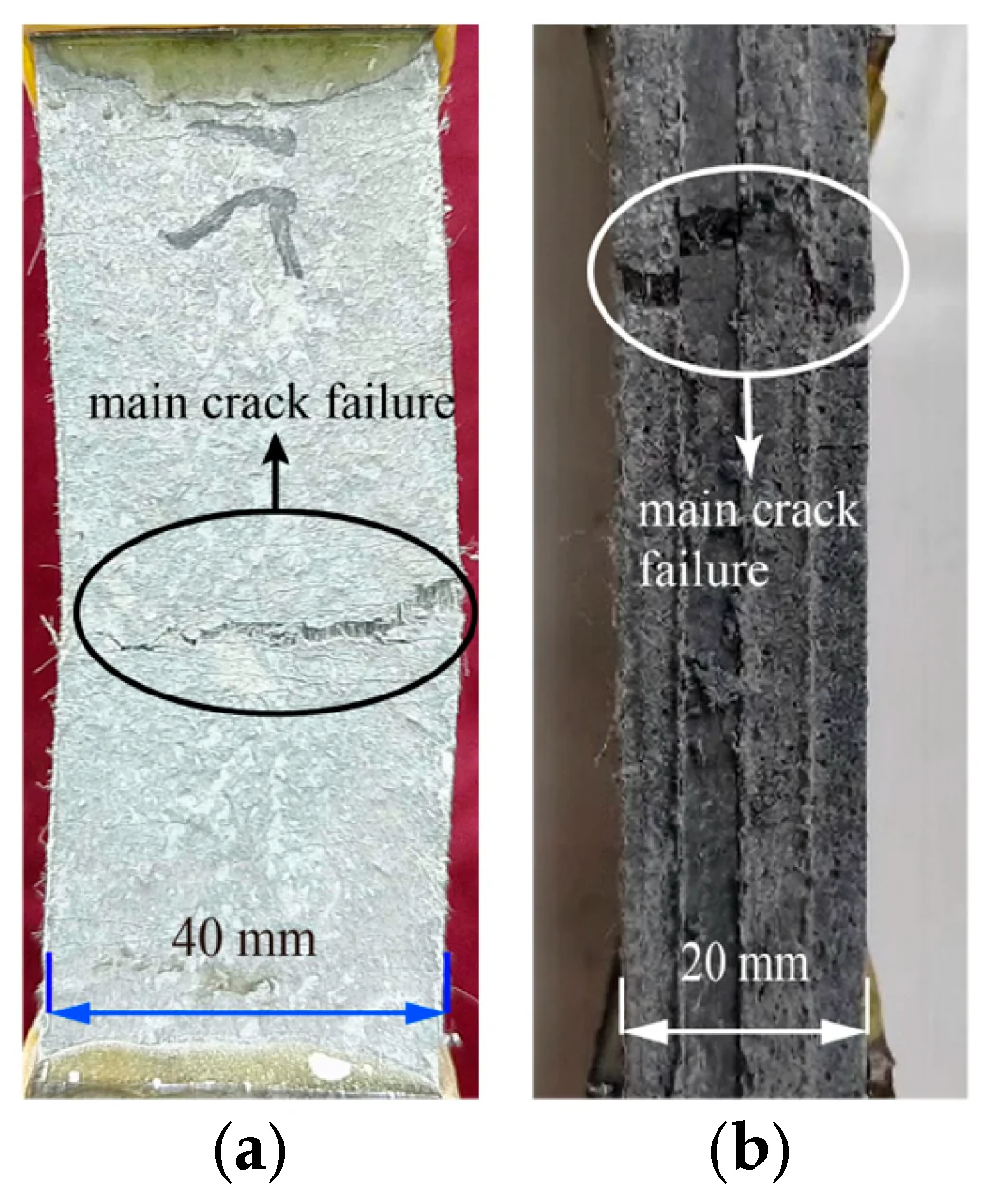
Failure mode of BTR-ECC at 15° off-axis angle: (**a**) front view; (**b**) side view.

**Figure 8:**
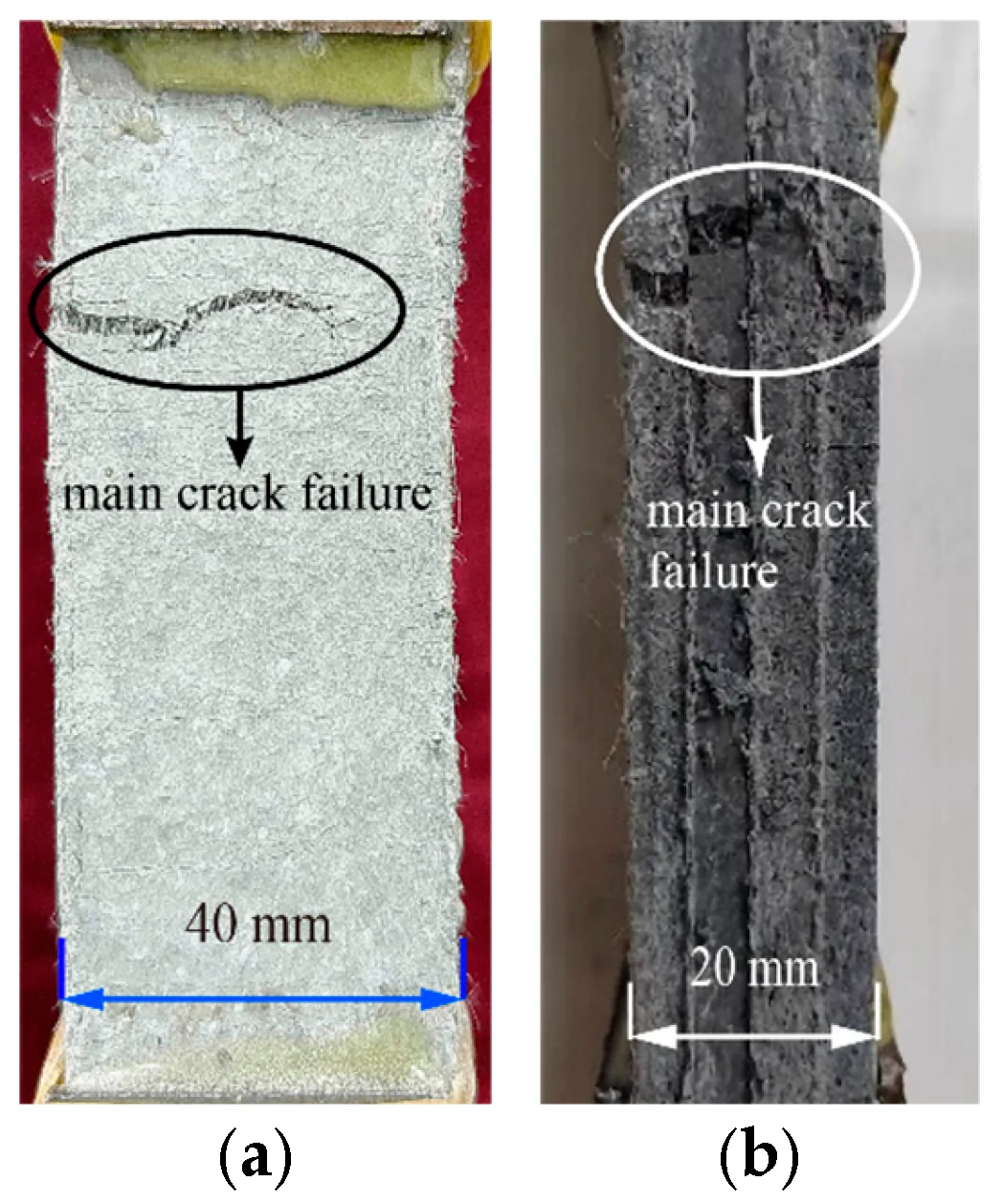
Failure mode of BTR-ECC at 30° off-axis angle: (**a**) front view; (**b**) side view.

**Figure 9:**
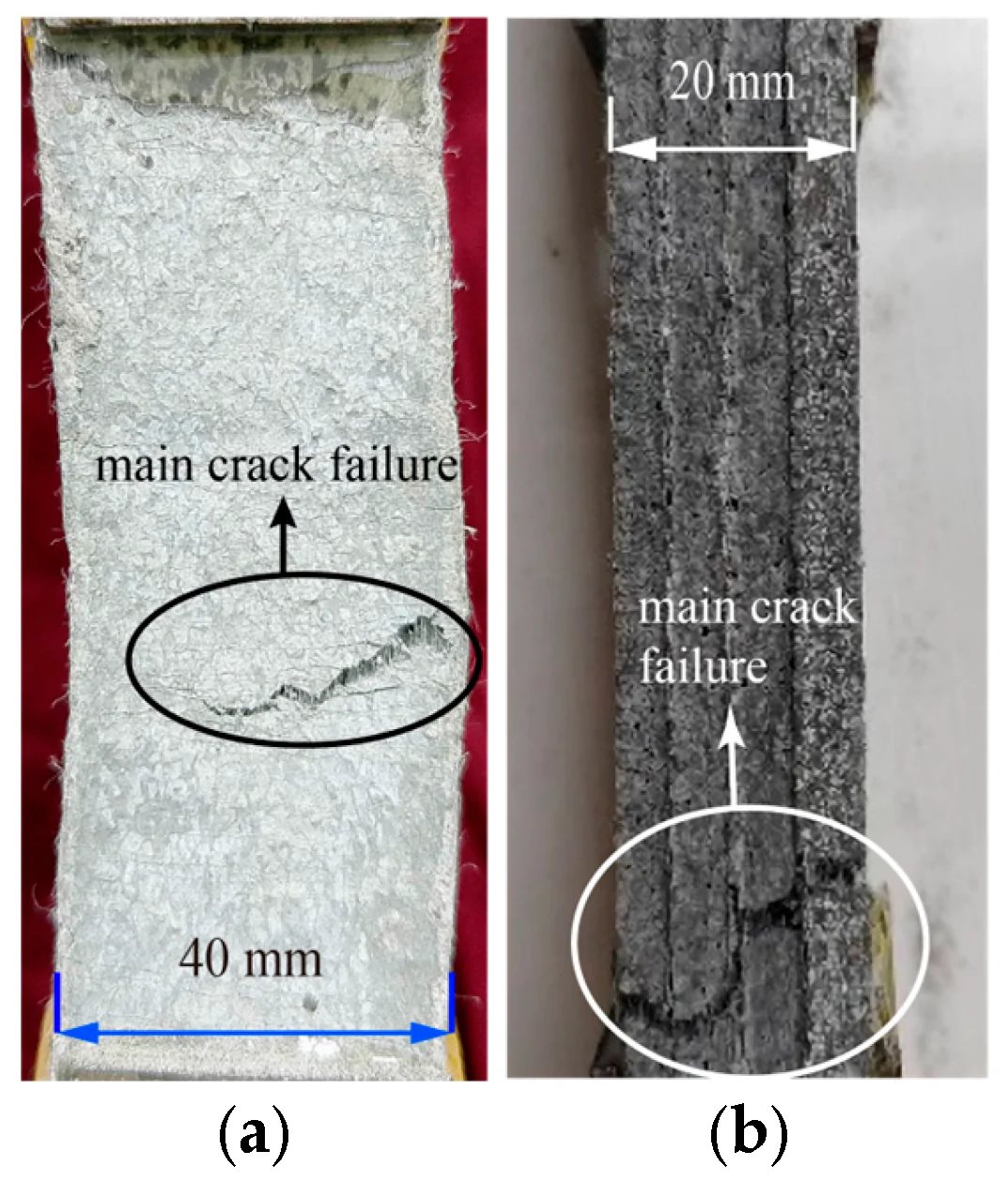
Failure mode of BTR-ECC at 45° off-axis angle: (**a**) front view; (**b**) side view.

**Figure 10:**
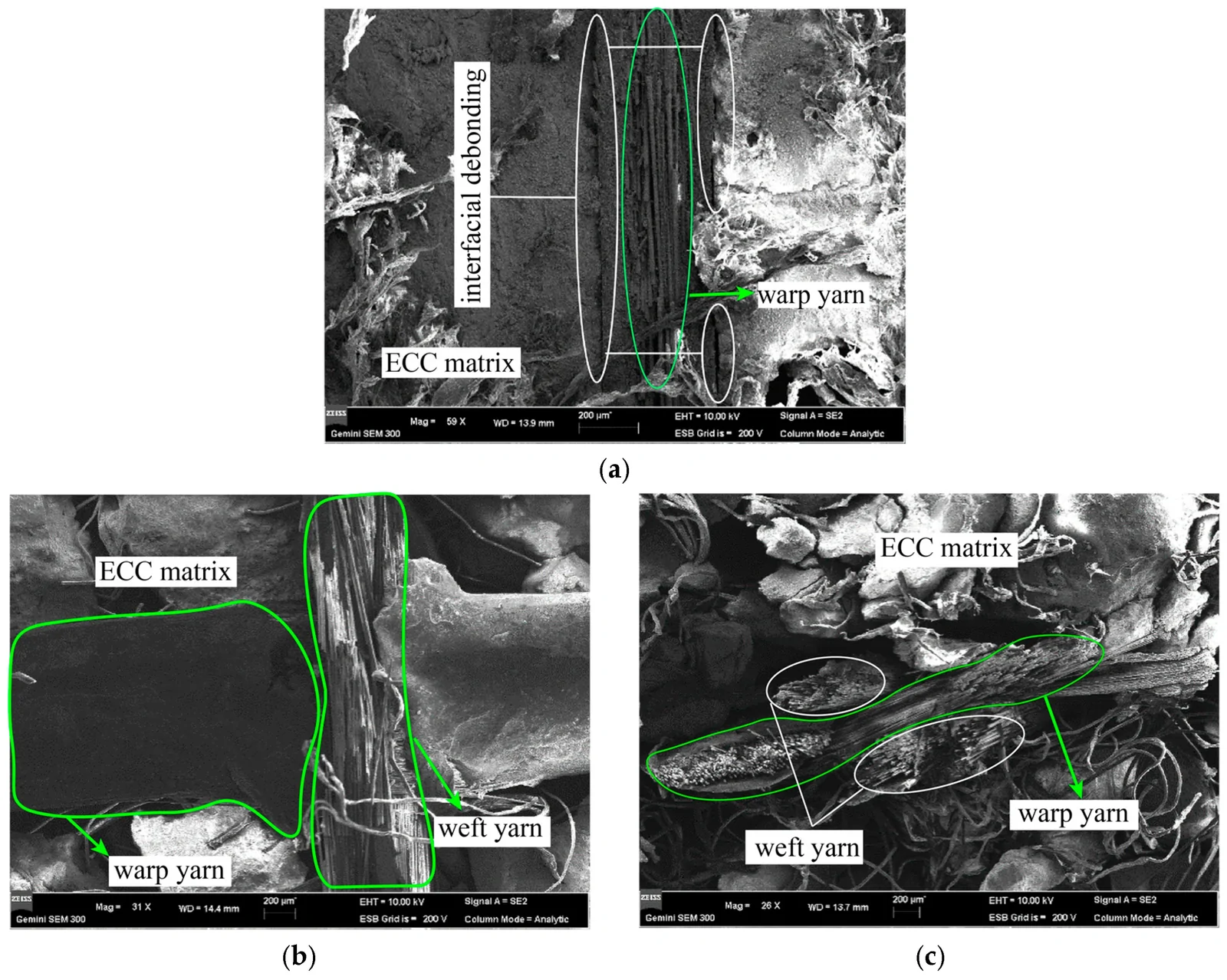
SEM images of failure morphology of BTR-ECC specimens: (**a**) Interface debonding between yarns and matrix of the 0° case; (**b**) Yarn pull-out of the 45° case; (**c**) Crack cross-section of the 45° case.

Figure 6, Figure 7, Figure 8 and Figure 9 present the macroscopic failure morphologies and surface crack distributions of BTR-ECC under tensile loading at different off-axis angles. For SEM observation, specimens at 0° and 45° off-axis angles were prepared separately (five per case). At 0°, samples were cut along the loading direction to observe the fiber bundle–ECC interface; at 45°, samples were taken from the main crack region to examine the failure morphology. All samples were ultrasonically cleaned in ethanol, dried, and gold-coated (~5 nm). Imaging was performed on a Gemini SEM 300 manufactured by Carl Zeiss Microscopy Ltd., Oberkochen, Germany in high vacuum mode (<5 × 10^−4^ Pa) using a secondary electron detector (SE2) at an accelerating voltage of 10 kV, and representative images were selected for analysis. Figure 10 shows the SEM images.

All BTR-ECC specimens did not fracture completely when reaching the ultimate strength, and a large number of short fibers were observed bridging the main crack, indicating that the strain-hardening characteristics of the ECC were effectively utilized. However, there is a distinct difference in crack propagation paths between axial and off-axis loading. Under 0° loading, the main crack propagated in a nearly symmetric manner from the middle of the specimen (Figure 6a), representing a typical tensile failure mode. In contrast, under off-axis loading, the main crack generally initiated from one side and extended toward the opposite side, exhibiting an asymmetric flexural cracking feature (Figure 7a, Figure 8a and Figure 9a). This difference may arise from the inherent anisotropy of the textile-reinforced composite. Owing to the differences in mechanical properties and spatial weaving configurations between the warp and weft yarns within the basalt-fiber textile, the magnitudes and directions of stresses carried by the warp and weft yarns undergo non-uniform redistribution under off-axis loading, thereby inducing in-plane asymmetric stress concentrations, which eventually cause cracks to initiate at local weak zones and propagate along specific paths.

From the side failure morphologies (Figure 6b, Figure 7b, Figure 8b and Figure 9b), it can be observed that the ECC matrix and the basalt-fiber textile maintained good integrity between layers at all off-axis angles, without apparent interlayer debonding or interfacial delamination. This suggests that in this composite system, the tensile strength of the basalt-fiber textile is relatively limited, and its own strength appears to be insufficient to overcome the toughness resistance of the ECC matrix and the bonding at the yarn/matrix interface, thus not leading to interlayer separation. The failure energy is likely dissipated primarily through multiple cracking of the matrix, fiber bridging, and yarn pull-out.

The failure modes of BTR-ECC differ markedly from those of GTR-ECC and CTR-ECC. Compared with GTR-ECC, which exhibits quasi-ductile failure that remains consistent across all off-axis angles, BTR-ECC also undergoes quasi-ductile failure but transitions from symmetric failure at 0° to asymmetric failure at off-axis angles. In contrast, CTR-ECC transitions from brittle failure at 0° to delamination at other off-axis angles. Notably, neither GTR-ECC nor BTR-ECC exhibits delamination under any loading condition.

#### 3.2.2. Crack Analysis

The crack openings are very fine across all angles (Figure 6a, Figure 7a, Figure 8a and Figure 9a), indicating that the BTR-ECC exhibits good crack control capability. At the peak stress, none of the specimens fractured completely, and a large number of pulled-out PVA fibers were visible in the crack regions, indicating the bridging effect of the ECC matrix.

Based on the analysis of surface images of the specimens after failure, the average crack opening and spacing were estimated using Equations (5) and (6), and the results are presented in Table 5. Equations (5) and (6) are taken from Zhu et al. [38].

(5) $$l^{cra}=\frac{L^{gau}}{n-1}$$

(6) $$w^{cra}=\frac{\varepsilon^{pe}-\varepsilon^{cra}}{n}L^{gau}$$

where $l^{\mathrm{cra}}$ is the estimated average crack spacing, $w^{\mathrm{cra}}$ is the estimated average crack opening, $L^{\mathrm{gau}}$ is the gauge length, n is the number of cracks within the gauge length, $\varepsilon^{\mathrm{pe}}$ is the strain corresponding to the peak strength, and $\varepsilon^{\mathrm{cra}}$ is the first-cracking strain. Since crack initiation is a progressive process without a distinct point in time, this study adopted the point where noticeable fluctuations begin on the average stress–strain curve as the first-cracking strain.

The indirect estimation method given as Equations (5) and (6) adopts a uniform crack distribution assumption and a uniform strain distribution assumption between adjacent cracks, which are reasonable for approximating the overall crack morphology to analyze evolution trends. For an independent comparison, high-resolution images were used to randomly select ten cracks from each specimen for pixel-based measurement, as also listed in Table 5. The image-based method yields substantially higher CMAD values than the indirect method, reflecting the inherent localized variability of crack patterns in ECC materials. The mean values from both methods are of the same order of magnitude, corroborating that the indirect method provides physically reasonable estimates of the overall average crack morphology.

Given the high uncertainty of the image-based method discussed above, the results obtained from Equations (5) and (6) are employed for crack analysis. As shown in Table 5, the estimated average crack openings at all off-axis angles are controlled within the range of 30–50 μm, indicating that BTR-ECC retains the favorable crack control capability of ECC [39]. The estimated average crack spacing from Equation (5) ranges from 2.1 mm at 0° to 1.4 mm at 45°, with coefficients of variation between 2.4% and 6.3%. Both the crack opening and crack spacing remain within the same order of magnitude across all tested angles. Considering the substantial scatter of the image-based measurements, no distinct trend with off-axis angle can be identified for the crack morphology. Therefore, BTR-ECC provides effective and consistent crack control across all off-axis angles.

Compared to GTR-ECC and CTR-ECC, BTR-ECC exhibits a similar crack spacing, all ranging from 1 to 2 mm. This may be attributed to the similar mesh sizes of the three types of textiles. However, the estimated average crack opening of BTR-ECC (30–50 μm) is narrower than that of both GTR-ECC (60–120 μm) and CTR-ECC (60–100 μm), indicating that the basalt textile may possess a superior stress-transfer capacity compared to the AR-glass and carbon textiles, which can be attributed to the better interfacial compatibility between the basalt textile and the ECC matrix.

#### 3.2.3. Failure Mechanisms

Under axial tension (0°), after crack initiation, the load is primarily sustained by the basalt-fiber textile. Relying on its relatively high tensile strength, the yarns can continuously carry the load, allowing the stress–strain curve to maintain an approximately linear ascending trend after cracking, with the serrated fluctuations during this stage likely resulting from ongoing micro-cracking of the ECC matrix. As the load further increases, the yarns gradually reach their tensile strength and fracture progressively one by one. SEM observations of the fracture surfaces (Figure 10a) further corroborate this interpretation. After loading, apparent debonding damage appears at the interface. By this stage, the load transfer path appears to have largely shifted to the textile, consistent with the macroscopic observation that load is mainly carried by the textile after cracking. In addition, the good interfacial bonding between yarns and the matrix, as well as the mechanical anchoring effect provided by interwoven warp and weft yarns, presumably contributes to a high pull-out resistance of fractured yarns, allowing partial residual load-bearing capacity to be retained. Consequently, the stress–strain curve exhibits a gradual descending trend after the peak rather than dropping abruptly.

Under off-axis tension, after matrix cracking the load is primarily carried by the basalt-fiber textile, and the yarns at crack locations are presumably subjected to combined tension and bending loading. SEM observations (Figure 10b,c) provide mesoscopic evidence supporting this combined effect. Specifically, under 45° loading, apparent slip and pull-out of yarns are observed, with significant separation between yarns and the ECC matrix at fracture surfaces, and yarns exhibit a failure morphology suggestive of combined tension-bending effects. As the load increases, the relatively high strength of basalt fibers presumably enables the textile to continue carrying load under off-axis conditions. It is inferred that before the interfacial shear stress reaches the critical value required to trigger interlayer delamination, the yarns are likely to have already fractured or pulled out. Consequently, BTR-ECC avoids delamination failure and maintains good deformation compatibility across all off-axis angles, while retaining a substantial strength enhancement over the plain matrix. In comparison, the strength improvement in GTR-ECC is presumably limited by the lower reinforcing capacity of the AR-glass textile, whereas CTR-ECC may suffer from relatively weak textile–matrix interfacial properties. In CTR-ECC, the interlayer shear stress generated by yarn pull-out could exceed the tolerance of the ECC matrix, leading to delamination failure rather than the synergistic global failure observed in BTR-ECC.

## 4. Phenomenological Constitutive Model

### 4.1. Model Development

Based on the characteristics of the off-axis tensile stress–strain curves of BTR-ECC shown in Figure 5, the mechanical response can be clearly divided into a linear elastic stage and a nonlinear hardening stage. The slopes of the initial elastic stage, or the elastic modulus, vary little across different off-axis angles, while the main distinction lies in the evolution of the nonlinear stage. Therefore, a piecewise function model is established to describe the stress–strain relationship under various off-axis angles, as given in Equation (7).

(7) $$\sigma^{\alpha }=\{\begin{array}{cc} E^{\alpha e}\varepsilon^{\alpha }, & 0<\varepsilon^{\alpha }<\varepsilon^{\alpha e}\\ \sigma^{\alpha d}(\varepsilon^{\alpha d},\alpha )+E^{\alpha e}\varepsilon^{\alpha e}, & \varepsilon^{\alpha e}\leq \varepsilon^{\alpha }<\varepsilon^{\alpha u} \end{array}$$

where $\sigma^{\alpha }$ and $\varepsilon^{\alpha }$ represent the stress and strain of BTR-ECC at off-axis angle α, respectively, with α=0°,15°,30°,45°; $\varepsilon^{\alpha e}$ is the elastic limit strain of BTR-ECC under the off-axis angle α; $\varepsilon^{\alpha u}$ is the ultimate strain of BTR-ECC under the off-axis angle α; $E^{\alpha e}$ is the elastic modulus of BTR-ECC in the linear stage under the off-axis angle α; $\sigma^{\alpha d}\left(\varepsilon^{\alpha },\alpha \right)$ is the stress component in the nonlinear stage, obtained by subtracting the critical elastic stress $E^{\alpha e}\varepsilon^{\alpha }$ from the total stress $\sigma^{\alpha }$.

For all loading cases, $\varepsilon^{\alpha e}$ and $E^{\alpha e}\varepsilon^{\alpha e}$ are taken as 0.2% and 4 MPa, respectively, based on the measured curves. This uniform setting is justified because the first-cracking properties of BTR-ECC show insignificant differences between on-axis and off-axis tension, as elaborated in the discussion of first-cracking stress. Moreover, due to fluctuations in the experimental curves at the first-cracking stage, the exact first-cracking point under off-axis loading is difficult to pinpoint, and no clear angle-dependent trend is observed. Therefore, using a single set of first-cracking parameters for all angles has a limited influence on the simulated stress–strain response and is acceptable for engineering practice.

To determine the nonlinear function $\sigma^{\alpha d}\left(\varepsilon^{\alpha },\alpha \right)$, the tangent modulus method is adopted as shown in Equation (8). The tangent modulus $E^{\alpha T}$ is defined as the instantaneous slope of the stress–strain curve at a given point:

(8) $$E^{\alpha T}=\frac{d\sigma^{\alpha }}{d\varepsilon^{\alpha }}$$

For discrete experimental data points, the central difference method approximates the tangent modulus $E^{\alpha Ti}$ at each point, as in Equation (9):

(9) $$E^{\alpha Ti}\approx \frac{\sigma^{\alpha (i+1)}-\sigma^{\alpha (i-1)}}{\varepsilon^{\alpha (i+1)}-\varepsilon^{\alpha (i-1)}}$$

Within the nonlinear stage, the nonlinear strain component is defined as $\varepsilon^{\alpha d}=\varepsilon^{\alpha }-\varepsilon_{e}^{\alpha }$. The stress $\sigma^{\alpha d}\left(\varepsilon^{\alpha d},\alpha \right)$ in this stage follows from integrating the tangent modulus $E^{\alpha Ti}$ across the corresponding strain range, as in Equation (10). The units are MPa for $\sigma^{\alpha d}$, GPa for $E^{\alpha T}$, and % for the strains $\varepsilon^{\alpha e}$, $\varepsilon^{\alpha u}$, and $\varepsilon^{\alpha d}$.

(10) $$\sigma^{\alpha d}(\varepsilon^{\alpha d},\alpha )=\int_{0}^{\varepsilon^{\alpha u}-\varepsilon^{\alpha e}\;}E^{\alpha T}\;d\varepsilon^{\alpha d}$$

**Figure 11:**
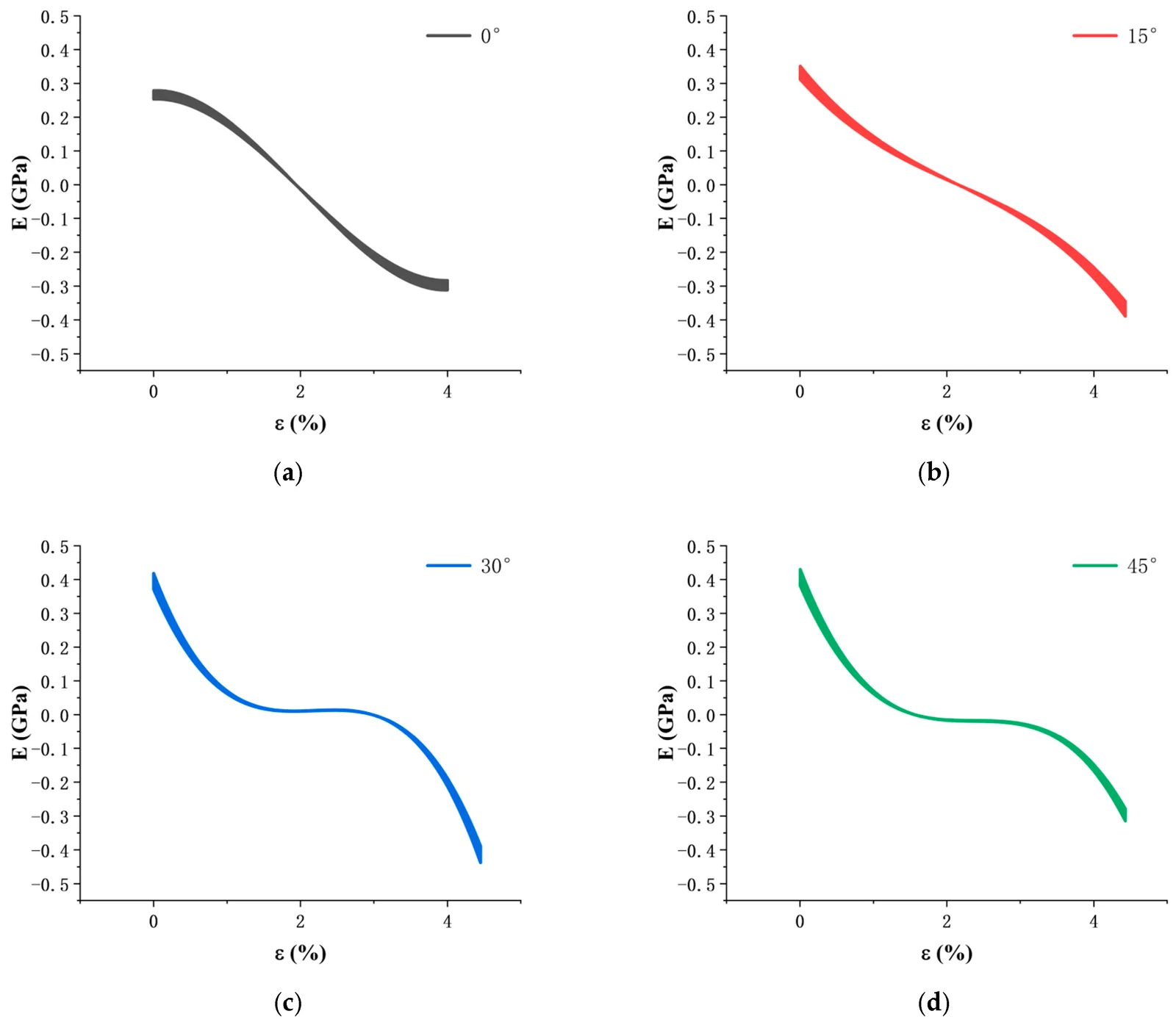
Tangent modulus versus strain at different off-axis angles: (**a**) 0°; (**b**) 15°; (**c**) 30°; (**d**) 45°.

Using Equations (8) and (9), the experimental data yield tangent modulus vs. strain curves for off-axis angles of 0°, 15°, 30°, and 45°, as shown in Figure 11.

Due to minor fluctuations from the high sampling frequency, the raw curves exhibit some thickness. To maintain data fidelity, no filtering or smoothing was applied. Figure 11 shows that the tangent modulus $E^{\alpha T}$ in the nonlinear stage at each angle drops rapidly at first and then levels off. This shape closely fits a cubic polynomial function. Therefore, the tangent modulus in this stage is assumed to follow a cubic polynomial function whose coefficients depend on the off-axis angle α, as written in Equation (11):

(11) $$E^{\alpha T}=a_{1}^{\alpha }{\left(\varepsilon^{\alpha d}\right)}^{3}+a_{2}^{\alpha }{\left(\varepsilon^{\alpha d}\right)}^{2}+a_{3}^{\alpha }\varepsilon^{\alpha d}+a_{4}^{\alpha }$$

Then, substituting Equations (10) and (11) into Equation (7) yields the complete constitutive model, which can be expressed by the following piecewise function.

(12) $$\sigma^{\alpha }=\{\begin{array}{cc} E^{\alpha e}\varepsilon^{\alpha }, & 0<\varepsilon^{\alpha }<\varepsilon^{\alpha e}\\ A_{1}^{\alpha }{\left(\varepsilon^{\alpha }-\varepsilon^{\alpha e}\right)}^{4}+A_{2}^{\alpha }{{\left(\varepsilon^{\alpha }-\varepsilon^{\alpha e}\right)}^{3}+A_{3}^{\alpha }{\left(\varepsilon^{\alpha }-\varepsilon^{\alpha e}\right)}^{2}+A_{4}^{\alpha }(\varepsilon^{\alpha }-\varepsilon^{\alpha e})+E}^{\alpha e}\varepsilon^{\alpha e}, & \varepsilon^{\alpha e}\leq \varepsilon^{\alpha }<\varepsilon^{\alpha u} \end{array}$$

### 4.2. Model Goodness-of-Fit Assessment

For each test angle ($\alpha =0^{\circ },15^{\circ },30^{\circ },45^{\circ }$), the coefficients in Equation (11) were determined by fitting the stress–strain curves at the corresponding off-axis angles. The units of $E^{\alpha T}$ and $\varepsilon^{\alpha d}$ are GPa and %, respectively. Table 6 lists the fitted coefficients for each off-axis angle.

**Figure 12:**
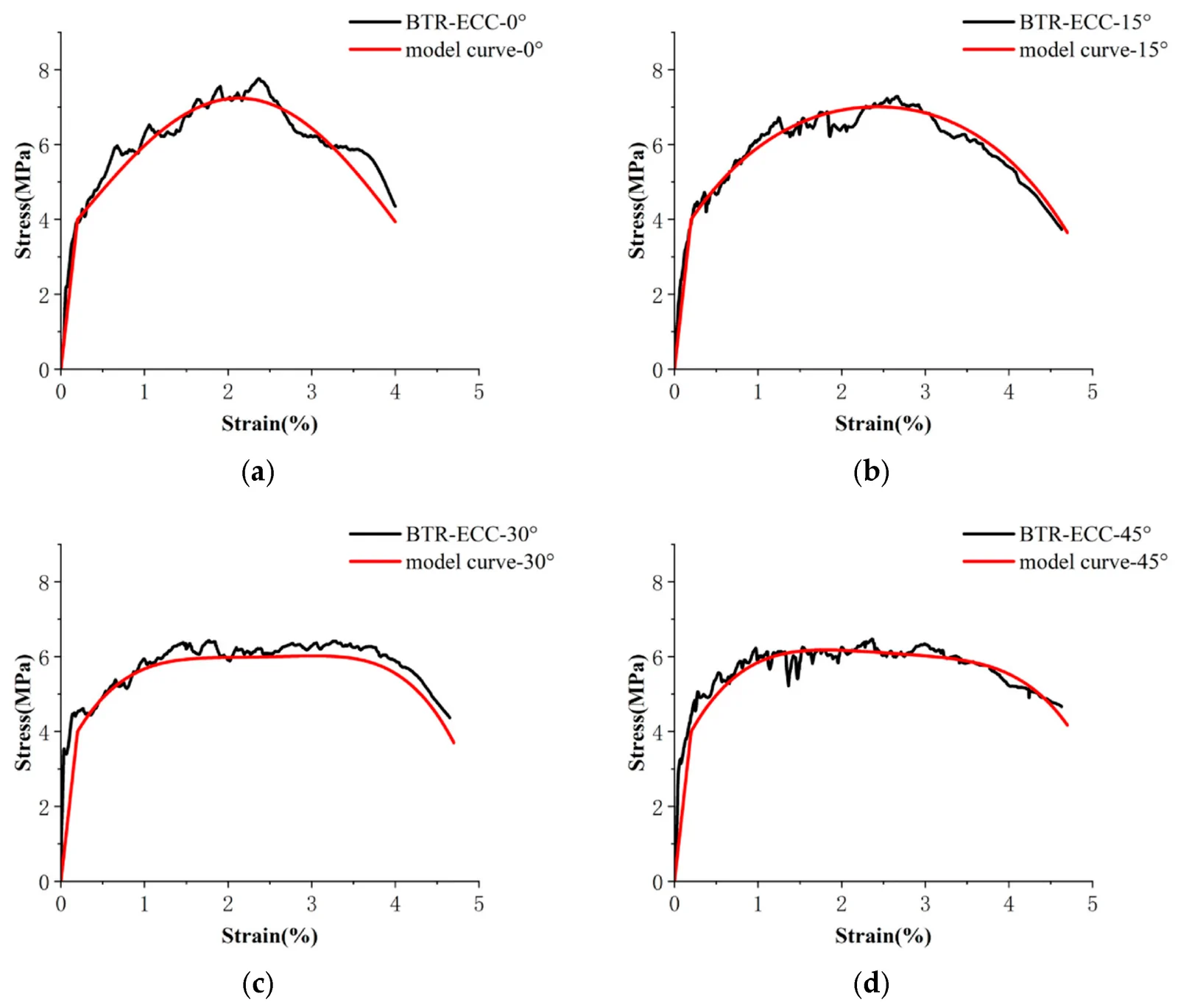
Comparison of model curves with experimental curves: (**a**) 0°; (**b**) 15°; (**c**) 30°; (**d**) 45°.

Once the coefficients $a_{i}^{\alpha }$ in Equation (11) were determined, the coefficients $A_{i}^{\alpha }$ in Equation (12) can be obtained by integration. The calculated coefficients $A_{i}^{\alpha }$ are summarized in Table 7. Subsequently, the model-derived stress–strain curves were plotted and compared to the experimental average curves, as shown in Figure 12. Furthermore, the coefficients of determination ($R^{2}$) and the global CMAD between the fitted curves and the experimental mean curves are given in Table 8.

Figure 12 indicates that the proposed piecewise constitutive model, established on the basis of the tangent modulus method, effectively captures the strain-hardening characteristics of BTR-ECC. The predicted peak strengths at off-axis angles of 0°, 15°, 30°, and 45° are 7.24 MPa, 7.01 MPa, 6.02 MPa, and 6.18 MPa, respectively, with relative errors ranging from −6.7% to −3.8% compared to the experimental data (Table 8). For the 0° and 15° cases, the $R^{2}$ values are above 0.9. For the 30° and 45° cases, although the $R^{2}$ values are slightly lower than 0.9, the global CMAD remains below 10%, indicating that the model provides an acceptable fitting quality within the calibrated range. These results demonstrate that the model is capable of describing the tensile mechanical behavior of BTR-ECC under different off-axis angles.

It should be noted that the proposed constitutive model is an empirical descriptive fit calibrated for BTR-ECC within the tested off-axis angles (0°, 15°, 30°, and 45°). While it should not be extrapolated to other material systems or untested orientations without further validation, the model provides a practical and efficient tool for rapidly describing the mechanical behavior of BTR-ECC in engineering design and finite element analysis. Furthermore, the established framework offers a useful reference for future investigations on similar textile-reinforced composites and intermediate off-axis angles.

## 5. Conclusions

(1) Variation in mechanical properties: The tensile strength of BTR-ECC at each off-axis angle is higher than that of plain ECC, but it exhibits a nonlinear reduction with increasing off-axis angle. Compared to the 0° direction, the tensile strengths at 15°, 30°, and 45° off-axis angles decrease by 6.1%, 17.3%, and 16.6%, respectively. The strength reduction may be associated with the altered load-bearing mechanism of the textile under off-axis loading, where the tensile capacity of the fibers is no longer fully mobilized.
(2) Failure modes and crack development: The failure of BTR-ECC material is associated with the combined action of textile fracture, yarn slip and pull-out, and PVA fiber bridging. No delamination is observed. For all loading cases, the estimated crack opening remains consistently within the range of 30–50 μm, suggesting good crack control capability under multi-directional loading.
(3) Phenomenological constitutive model: Based on the tangent modulus method, a piecewise constitutive model is established. This model provides a reasonable description of the full mechanical response of the material under different off-axis angles, and the fitted curves show good agreement with the experimental mean curves. The model may serve as a reference for engineering design and numerical analysis within the calibrated range of the four tested angles. However, further experimental validation is needed to confirm its applicability to other ECC mixtures, textile types, or intermediate off-axis angles beyond the tested range.
(4) Comparison with GTR-ECC and CTR-ECC: Relative to GTR-ECC and CTR-ECC, BTR-ECC exhibits distinct trends in strength, strain, energy density, and failure modes. This highlights the critical role of textile type in governing TR-ECC mechanics and confirms that the behavior of BTR-ECC cannot be simply inferred from previous studies on glass or carbon textiles.
(5) Design implication: The 0° lay-up is recommended for strength-critical applications and remains a viable, well-balanced option for deformation- or energy-critical designs, as its strain energy density stays nearly uniform across all angles despite a marginally lower ultimate strain at 0°.

## Figures and Tables

**Table 1:** Chemical composition of the premixed mortar.

No.	Test Item	Unit	Test Result
1	Loss on ignition	%	18.92
2	SiO_2_	%	21.57
3	CaO	%	43.87
4	Fe_2_O_3_	%	2.30
5	Al_2_O_3_	%	7.20
6	MgO	%	2.88
7	K_2_O	%	0.46
8	Na_2_O	%	0.16
9	Alkali content	%	0.46
10	SO_3_	%	1.49
11	Insoluble residue	%	14.51
12	Chloride	%	0.034
13	Density	g/cm^3^	2.80
14	Specific surface area	m^2^/kg	401

**Table 2:** Physical and mechanical parameters of PVA short fibers.

Tensile Strength (MPa)	Elastic Modulus (GPa)	Density (g/cm^3^)	Elongation at Break (%)	Length (mm)	Aspect Ratio	Diameter (μm)
494	15	1.25–1.35	3.1	16.5	600	27.5

**Table 3:** Mix proportion of ECC matrix components.

Component	Premixed Mortar Powder	Water	Superplasticizer	PVA Fibers
Qty. per batch (g)	600	108	6	4.8

**Table 4:** Mechanical property parameters of BTR-ECC.

Off-Axis Angle		0°	15°	30°	45°
First-cracking Stress (MPa)	BTR-ECC	3.92 (2.3%)	4.2 (19.1%)	4.18 (3.7%)	4.59 (14.6%)
	ECC	3.07	3.07	3.07	3.07
	Var. to ECC	27.7%	36.8%	36.2%	49.5%
	Var. to 0°	—	7.14%	6.63%	17.1%
Peak Strength (MPa)	BTR-ECC	7.76 (3.1%)	7.29 (6.0%)	6.42 (3.5%)	6.47 (5.9%)
	ECC	4.2	4.2	4.2	4.2
	Var. to ECC	84.8%	73.6%	52.9%	54.0%
	Var. to 0°	—	−6.1%	−17.3%	−16.6%
Ultimate Strain (%)	BTR-ECC	3.59 (0.3%)	3.58 (5.8%)	3.82 (2.9%)	3.79 (8.2%)
	ECC	7.56	7.56	7.56	7.56
	Var. to ECC	−68.8%	−64.7%	−50.3%	−57.5%
	Var. to 0°	—	13.1%	59.3%	36.0%
Strain Energy Density at failure ($kJ/m^{3}$)	BTR-ECC	220 (0.2%)	217 (5.4%)	223 (2.0%)	217 (12.6%)
	ECC	278	278	278	278
	Var. to ECC	−20.8%	−21.9%	−19.8%	−21.9%
	Var. to 0°	—	−1.4%	1.4%	−1.4%

Remark: The values in parentheses are the CMADs for the corresponding parameters.

**Table 5:** Crack parameters of BTR-ECC.

	0°	15°	30°	45°
Estimated average crack opening (Equation (6))	49 μm (21%)	42 μm (21%)	39 μm (19%)	30 μm (26%)
Estimated average crack opening (image)	56 μm (36%)	50 μm (40%)	42 μm (35.2%)	30 μm (53.3%)
Estimated average crack spacing (Equation (5))	2.1 mm (6.3%)	1.9 mm (5.3%)	1.7 mm (5.9%)	1.4mm (2.4%)
Estimated average crack spacing (image)	1.6 mm (58.4%)	1.5 mm (60.1%)	1.2 mm (37%)	1.6 mm (53%)
Estimated average crack counts	49 (7.5%)	54 (6.2%)	62 (5.9%)	71 (4.2%)

Remark: The values in parentheses are the CMADs for the corresponding parameters.

**Table 6:** Results of fitting coefficients.

Off-Axis Angle	$a_{1}^{\alpha }$	$a_{2}^{\alpha }$	$a_{3}^{\alpha }$	$a_{4}^{\alpha }$
0°	0.186	−1.12	0.101	2.66
15°	−0.108	0.699	−2.48	3.19
30°	−0.366	2.40	−5.17	3.76
45°	−0.301	2.15	−5.12	3.91

**Table 7:** Summary of coefficients A for the complete model.

Off-Axis Angle α	0°	15°	30°	45°
$A_{1}^{\alpha }$	0.0465	−0.027	−0.0915	−0.0753
$A_{2}^{\alpha }$	−0.373	0.233	0.8	0.717
$A_{3}^{\alpha }$	0.0505	−1.24	−2.59	−2.56
$A_{4}^{\alpha }$	2.66	3.19	3.76	3.91

**Table 8:** Goodness-of-fit assessment.

Off-Axis Angle	0°	15°	30°	45°
Peak Strength (Tested) (MPa)	7.76	7.29	6.42	6.47
Peak Strength (Model) (MPa)	7.24	7.01	6.02	6.18
Peak Strength Error	−6.7%	−3.8%	−6.2%	−4.5%
R^2^	0.92	0.96	0.73	0.81
Global CMAD	6.47%	4.80%	6.79%	4.48%

## Data Availability

The original contributions presented in this study are included in the article material. Further inquiries can be directed to the corresponding author.
